# *Neurospora* WC-1 Recruits SWI/SNF to Remodel *frequency* and Initiate a Circadian Cycle

**DOI:** 10.1371/journal.pgen.1004599

**Published:** 2014-09-25

**Authors:** Bin Wang, Arminja N. Kettenbach, Scott A. Gerber, Jennifer J. Loros, Jay C. Dunlap

**Affiliations:** 1 Department of Genetics, Geisel School of Medicine, Dartmouth, Hanover, New Hampshire, United States of America; 2 Norris Cotton Cancer Center, Geisel School of Medicine, Dartmouth, Hanover, New Hampshire, United States of America; 3 Department of Biochemistry, Geisel School of Medicine, Dartmouth, Hanover, New Hampshire, United States of America; Charité - Universitätsmedizin Berlin, Germany

## Abstract

In the negative feedback loop comprising the *Neurospora* circadian oscillator, the White Collar Complex (WCC) formed from White Collar-1 (WC-1) and White Collar-2 (WC-2) drives transcription of the circadian pacemaker gene *frequency* (*frq*). Although FRQ-dependent repression of WCC has been extensively studied, the mechanism by which the WCC initiates a circadian cycle remains elusive. Structure/function analysis of WC-1 eliminated domains previously thought to transactivate *frq* expression but instead identified amino acids 100–200 as essential for *frq* circadian expression. A proteomics-based search for coactivators with WCC uncovered the SWI/SNF (SWItch/Sucrose NonFermentable) complex: SWI/SNF interacts with WCC in vivo and in vitro, binds to the *Clock box* in the *frq* promoter, and is required both for circadian remodeling of nucleosomes at *frq* and for rhythmic *frq* expression; interestingly, SWI/SNF is not required for light-induced *frq* expression. These data suggest a model in which WC-1 recruits SWI/SNF to remodel and loop chromatin at *frq*, thereby activating *frq* expression to initiate the circadian cycle.

## Author Summary

Circadian clocks govern behavior in a wide variety of organisms. These clocks are assembled in such a way that proteins encoded by a few dedicated “clock genes” form a complex that acts to reduce their own expression. That is, the genes and proteins participate in a negative feedback loop, and so long as the feedback has delays built in, this system will oscillate. The feedback loops that underlie circadian rhythms in fungi and animals are quite similar in many ways, and while much is known about the proteins themselves, both those that activate the dedicated clock genes and the clock proteins that repress their own expression, relatively little is known about how the initial expression of the clock genes is activated. In *Neurospora*, a fungal model for these clocks, the proteins that activate expression of the clock gene “*frequency*” bind to DNA far away from where the coding part of the gene begins, and a mystery has been how this action-at-a-distance works. The answer revealed here is that the activating proteins recruit other proteins to unwrap the DNA and bring the distal site close to the place where the coding part of the gene begins.

## Introduction

Circadian clocks are key cellular mechanisms regulating a wide variety of physiological and molecular activities. *Neurospora* has been for several decades an excellent model for studies of the eukaryotic circadian clock characteristic of fungi and animals. In this organism, the White Collar Complex (WCC), a heterodimer comprised of WC-1 and WC-2, serves as the transcriptional activator for the pacemaker gene *frequency* (*frq*) by binding to one of two DNA elements, the *Clock box* (*C box*) [1] in the dark or the *Proximal Light-Response Element* (*PLRE*) in the light [2]. FRQ protein interacts with FRQ-Interacting RNA Helicase (FRH) to bring about repression of WCC activity and thereby to close the positive arm of the feedback loop [3]–[6], presumably through the phosphorylation of WCC [7], [8]. FRQ-mediated WCC repression has been extensively studied, whereas how WC-1 as a transcription factor drives *frq* expression in a circadian cycle is still poorly understood. WC-1 has two predicted transactivation domains located close to the N- and C- termini respectively [2], [9]–[11]. However, no experimental data have yet confirmed their *in vivo* functions.

Nucleosome and chromatin structure play critical roles in transcriptional regulation. To overcome nucleosomal barriers in transcription, different transcriptional complexes coordinate to make genomic DNA accessible to RNA polymerase II (reviewed by [12]. Two classes of chromatin remodeling enzymes have been shown to facilitate transcription of chromatin templates *in vivo*, the histone acetyltransferases and the ATP-dependent remodeling enzymes [13]. SWI/SNF (SWItch/Sucrose NonFermentable), one of the ATP-dependent chromatin remodeling complexes, was first discovered in *Saccharomyces cerevisiae* as a 2 MDa complex that alters chromatin structures for essential functions such as transcriptional activation, DNA repair, recombination, and chromosome segregation [14], [15]. In yeast SWI/SNF is estimated to control the transcription of no more than 5% of all genes but including the acid phosphatase genes and the MATα-Specific genes [16]. The yeast SWI/SNF complex contains one copy each of eleven subunits, except for the SWI3 subunit that is present in two copies; all SWI/SNF subunit-null mutants are viable but display distinguishable phenotypes. SWI2 is a highly conserved DNA-dependent ATPase and the scaffold protein of the complex [17]; *swi2* knockouts are defective in sporulation and display slow growth on nonfermentable carbon sources [18], [19]; *swi1* null strains, defective in mating-type switching, display sporulation defects and slow growth [20], [21]; *snf5* null mutants show reduced growth on glucose and sucrose [22], [23]. The SWI/SNF complex can associate with naked DNA or nucleosomes, and is thought not to bind in a sequence specific manner [24]–[26]. Instead, SWI/SNF is targeted to promoters via acidic domains on the surfaces of gene-specific transcriptional activators rather than via interactions with polyglutamine (polyQ) rich sequences [27]. Yeast SWI/SNF can alter nucleosomal structure in an ATP-dependent manner, which leads to the relief of chromatin-mediated repression of transcription [28], [29]. SWI/SNF is able to remodel nucleosomes *per se* without their disruption, by sliding histone octamers to other sites on the same DNA molecule, or transferring histone octamers to other DNA molecules [26].

Rhythmic histone modifications and chromatin remodeling over a circadian cycle have been reported for a variety of genes in different circadian systems. For example, the promoter regions of *Per1* and *Per2*, central clock components in mammals, undergo rhythmic histone H3 acetylation (K9, K14) [30]. In *Neurospora*, histone acetylation of the *frq* promoter oscillates over a circadian cycle and chromatin structure at *frq* is rhythmically remodeled in a circadian fashion, accompanied by rhythmic binding of WC-2 [31]. The occupancy of the nucleosome neighboring the *C box* peaks when *frq* transcription is repressed and decreases just before *frq* transcription starts [31]. In the same study, an ATP-dependent chromatin remodeler, CLOCKSWITCH (CSW), was identified as a component essential for depositing nucleosomes back to the *C box* to terminate *frq* transcription. Another chromatin remodeler, CHD-1, plays a role in methylation and rhythmic expression of *frq* [32]. Although both CSW and CHD-1 appear to be required for the closure of *frq* transcription and participate in chromatin remodeling at *frq*, how the WCC interacts with remodeling factors to relieve chromatin-mediated repression of the *C box* is still elusive. Consistent with this, there is a several hour lag between the turnover of FRQ and the rapid increase in *frq* mRNA [33], suggesting that there is more to reinitiation of *frq* expression than simply relief of repression.

In this study, we identify a previously undescribed N-terminal domain of WC-1, close to but not including the prominent polyglutamine stretch, that acts as the transactivation domain for recruiting SWI/SNF and driving *frq* transcription; notably, elimination of the N- and C- terminal polyQ stretches does not influence *frq* circadian transcription and such strains showed a WT circadian phenotype. The SWI1 subunit in the SWI/SNF complex is essential for initiation of *frq* transcription in a circadian context although interestingly not in response to light, and in a Δ*swi1* strain chromatin structure adjacent to the *C box* is less remodeled and the oscillation of the nucleosomal density is abolished, as is the circadian clock.

## Results

### N- and C-terminal polyQ stretches predicted to be activation domains are not required for WCC circadian function

**Figure 1:**
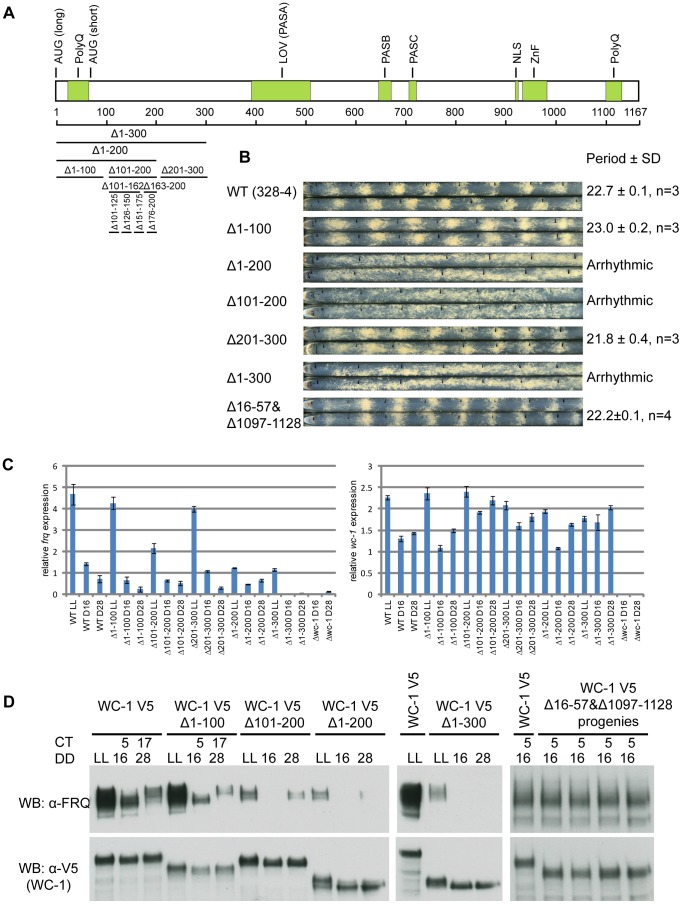
Identification of aa 100-200 as the transcriptional activation domain for WC-1. (**A**) Schematic depiction of the domain architecture of the WC-1 protein. WC-1 contains 1,167 amino acids and black bars with corresponding numbers of amino acids represent different deletions of the N-terminal region of WC-1. AUG(long) and AUG(short) mark the beginnings of long and short forms of WC-1; PolyQ, putative transcription activation domain; LOV, light, oxygen, or voltage domain containing FAD chromophore binding site; PAS, Per-Arnt-Sim domain mediating interaction with WC-2; NLS, putative nuclear localization signal; Zn, zinc finger DNA-binding domain (**B**) Racetube analyses of wild-type (WT) and WC-1 deletion strains. Period is reported in hours ± one standard deviation, n  =  number of racetubes. Replicate tubes are shown, and the vertical black lines in racetubes mark daily growth fronts of the strains. 328-4 (*ras-1^bd^ A*) served as the WT. Strains bearing deletions of both polyQs (Δ16-57 and 1097-1128), Δ1-100, and Δ201-300 showed WT circadian periods. Strains lacking aa 100-200 (Δ1-200, Δ1-300, and Δ101-200) are arrhythmic (**C**) Results from quantitative RT-PCR analysis of *frq* and *wc-1* mRNA levels in WT and in the *wc-1* deletion strains noted. (**D**) Western blot showing expression levels of WC-1 and FRQ in WT and *wc-1* mutants. LL, constant light; DD, hours after the light to dark transition; CT, circadian time. Both WT WC-1 and mutants were C-terminally tagged with V5. Δ101-200 showed a significant decrease of *frq* expression and further deletion of aa 1-200 or 1-300 further dimished *frq* expression. Elimination of the two polyQs (Δ16-57 and 1097-1128; four progeny of identical genetype from one cross are shown) had no impact on FRQ level at CT5.

In the classic model of transcription, transcription factors use their transactivation domains to recruit transcriptional coactivators, e.g. chromatin remodelers and histone acetyltransferases, to release the repressive state of promoter regions [26]. In *Neurospora*, WC-1 has two polyQ domains located at N- and C- termini respectively that were hypothesized to be transactivation domains (AD) [10], [11], [34] (Figure 1A). To test the role of the two polyQs in *frq* expression, we eliminated both of them (aa 16-57 and aa 1097-1128) (Figure S1A) and surprisingly the double deletion strain still showed a wild-type (WT) circadian phenotype (Figure 1B). We further checked FRQ levels in strains held in constant dark (DD) for 16 hours (Circadian Time (CT) 5), a time when newly synthesized FRQ appears in WT. Consistent with the race tube data, FRQ levels in the double deletion strain showed no observable difference from WT and the WC-1 level is comparable with WT (Figure 1D, right-most panel). Collectively, the two polyQ stretches on WC-1 do not influence the stability of WC-1 and are not required for *frq* transcription and the circadian function of WCC in the dark.

### A domain encompassing aa 100-200 in WC-1 is required for *frq* dark expression

Because, despite prediction, the two polyQ stretches on WC-1 are not transactivation domains, a series of WC-1 deletions were generated to identify the regions needed for *frq* transcription, and circadian phenotypes of these mutants were monitored by race tube assays (Figure 1A, 1B, and Figure S1B). Of these mutants, only strains bearing deletions overlapping aa 101-200 showed an arrhythmic circadian phenotype (Figure 1B). In the three mutants deleted for these residues, all of which lacked overt rhythmicity, *frq* mRNA levels were no longer rhythmic and were reduced to or below levels seen in the trough of the wild type rhythm (Figure 1C). New FRQ was not seen at DD16 (16 hours in darkness, circadian time (CT) 5 in the subjective morning when *frq* expression normally peaks) and only a weak FRQ band or no FRQ at all appeared at DD28 (subjective night, CT17) (Figure 1D); levels of *wc-1* mRNA and WC-1 protein were normal. This suggested that the region between aa 101 and 200 has the potential to transactivate *frq* expression, and to identify these residues four strains bearing smaller deletions were created at intervals of 25 amino acids: Δ100-125, Δ126-150, Δ151-175, and Δ176-200. The Δ100-125 and Δ151-175 strains showed a WT period while Δ126-150 and Δ176-200 displayed a period of 2 hours longer than WT (Figure S1B). Thus, it seems that multiple domains determine the expression level of the *frq* gene. Interestingly, the Δ163-200 strain that displayed a 25.5 hour period (Figure S1B) and reduced dark FRQ expression (Figure S1C) retained its responsivity to light for both *frq* mRNA and FRQ expression respectively (Figure S1D), indicating that this region may only mediate circadian *frq* expression.

### WCC interacts with SWI1 *in vivo* and *in vitro*

**Figure 2:**
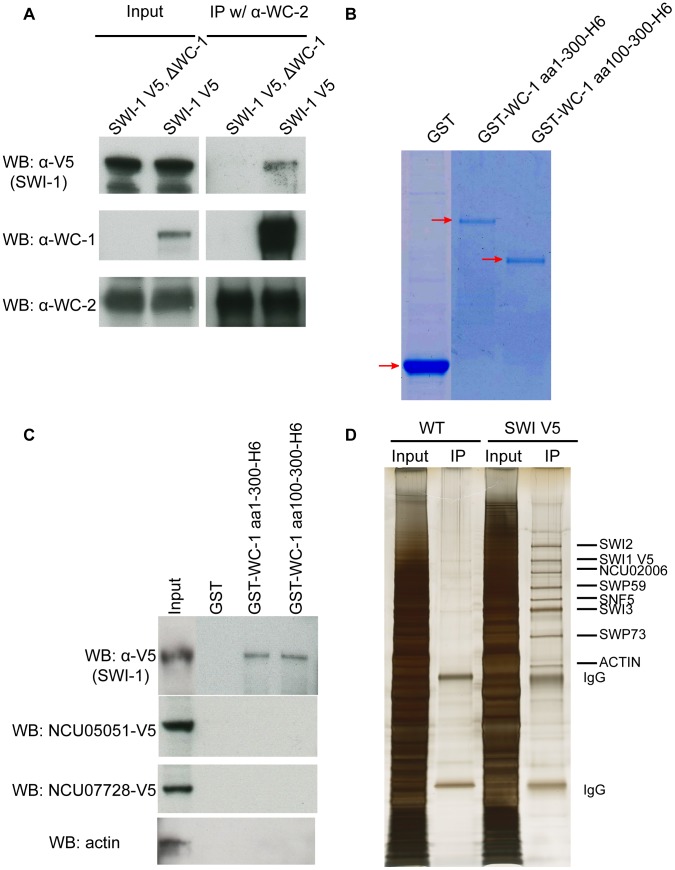
WCC interacts with SWI/SNF *in vivo* and *in vitro*. (**A**) Co-IP demonstrating interaction of WC-1 with SWI1 *in vivo*. SWI1 was C-terminally tagged with a V5 epitope and immunoprecipation was performed using WC-2 antibody. WC-1 was pulled down with WC-2 as well as a reduced amount of SWI1. (**B**) Expression and purification of GST-WC-1 fusion proteins. GST, GST WC-1 1-300 6xHis, and GST WC-1 100-300 6xHis lacking the N-polyQs were expressed in bacteria and purified. Gel stained with Coomassie Blue. (**C**) N-terminal fragments of WC-1 extending from 1-300 or 100-300 bind to SWI1 *in vitro*. Neurospora crude cell lysates were mixed with beads to which the GST-tagged WC-1 fragments were bound, and bound SWI1 was visualized by virtue of a C-terminal V5 tag; see Materials and Methods. GST alone failed to pull down SWI1 while WC-1 aa1-300 or aa 100-300 pulled down SWI1 at a similar level. Likewise negative control proteins actin, and two transcription factors encoded by NCU05051 and NCU07728 were not bound by WC-1 fragments. (**D**) Affinity purification of the *Neursopora* SWI/SNF complex showed the presence of different subunits in a 1∶1 stoichiometry except for SWI3. SWI1 was tagged with V5 at the C-terminus, centrifuged lyate was incubated with V5 antibody, and the gel was sliver-stained. Individual bands were excised and each protein identified via mass spectrometry.

To identify interaction partners of the WCC and potential co-activators, WC-1 that was epitope-tagged with V5, 10xhistidine, and 3xFLAG was purified from extracts and interacting proteins identified by MS/MS (Wang *et al*., in preparation); these preliminary mass spectrometry data showed that SWI/SNF subunits copurified with the *Neurospora* WCC. To validate this interaction *in vivo*, SWI1 was C-terminally tagged with V5. Immunoprecipitation using WC-2 antibody revealed the anticipated strong interaction with WC-1 as well as an interaction with SWI1 that was dependent on the presence of WC-1 (Figure 2A).

To confirm that the association between this region of WC-1 and SWI/SNF is still robust in vitro, GST alone, GST-WC-1 aa 1-300-6xHis and GST-aa 100-300-6xHis (that lacks the N-terminal polyQs) were cloned, expressed and purified from *Escherichia coli.* GST-WC-1 aa 1-300 6xHis and GST-aa 100-300-6xHis were purified with a two-step protocol to obtain the full length polypeptides (Figure 2B). To pull down SWI/SNF, the purified proteins were incubated with a centrifuged cell lysate of a SWI1-V5 strain and the captured proteins analyzed by Western blot. GST alone failed to pull down SWI1-V5 while GST-WC-1 aa 1-300-6xHis and GST-aa 100-300-6xHis pulled down SWI1 at a similar level (Figure 2C). Negative controls include actin, an abundant nuclear protein, as well as two transcription factors, the GATA Zn finger factor SRE encoded by NCU07728 and the Zn(2)-Cys(6) binuclear cluster domain transcription factor COL-23 encoded by NCU07728, both of which are known to bind DNA (X. Zhou and JCD, unpublished). The data indicate that WC-1 aa 1-300 is able to recruit SWI/SNF and aa 1-100 which contains the polyQs does not contribute to the recruitment. This is consistent with the behavior of yeast SWI/SNF that can be pulled down from crude cell lysates by the acidic transactivation domain but not by the polyQ region of herpes virus VP16 [27]. The isoelectronic point (pI) of the *Neurospora* N-terminal domain aa1-300 is 4.95 and aa 100-300 is 4.41 (predicted by DNAMAN software). The acidic nature of aa 100-300 is consistent with reported acidic activation domains of the VP16 protein, yeast Gcn4, and yeast Hap4, which are able to recruit the SWI/SNF complex to release chromatin-mediated repression of transcription [35]–[37].

To characterize the subunit composition of *Neurospora* SWI/SNF, V5-tagged SWI1 was purified using a single V5 antibody step and the result showed that several proteins were specifically co-purified with SWI1-V5 in a stoichiometric manner (Figure 2D). These bands were cut out individually and identified by mass spectrometry. The data confirmed the presence of SWI1, SWI2, SWI3, SWP59, SNF5, and SWP73 in the *Neurospora* SWI/SNF complex and confirmed that the interactions within complex in Neurospora are robust. Like WC-1 and WC-2, SWI1 and SWI2 have constant protein levels over 28 hours in constant dark (Figure S2). Taken together, these data demonstrate that WC-1 binds to SWI/SNF *in vivo* and WC-1 aa100-300 can specifically recruit SWI/SNF *in vitro*.

### Δ*swi1* and Δ*snf5* show significantly impaired *frq* expression

**Figure 3:**
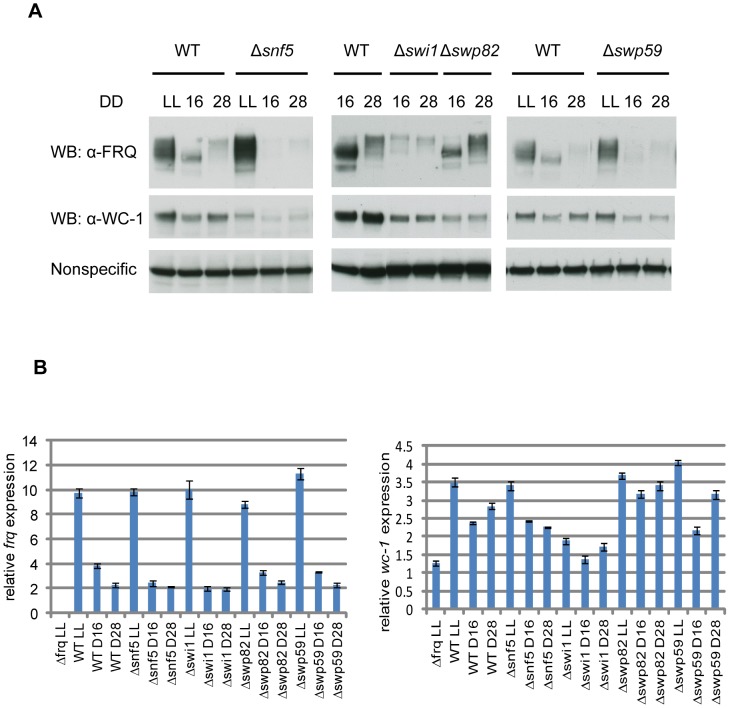
Loss of FRQ expression in SWI/SNF subunit knockouts. (**A**) Expression levels of FRQ and WC-1 were followed by Western blotting in WT, Δ*swp59*, Δ*swi1*, and Δ*snf5*. Two dark time points were chosen to examine FRQ expression, CT5 (DD16) when newly synthesized FRQ is seen and CT17 (DD28) when old FRQ is hyperphosphorylated and begins to be degraded in WT. Non-specific bands were shown for equal loading. (**B**) Corresponding data for *frq* and *wc-1* mRNA is shown.

To uncover a possible role of SWI/SNF in *frq* transcription and in circadian rhythmicity, we obtained all SWI/SNF single gene deletion strains from the *Neurospora* knockout collection (Colot *et al*., 2006) according to their homology with yeast SWI/SNF subunits (Figure 2D and Table 1) [38]. Except for SWI2 and SWI3 which may be essential for growth in minimal medium, all deletion strains of *Neurospora* SWI/SNF are viable, as are the knockouts of yeast SWI/SNF homologs (http://www.yeastgenome.org/). FRQ protein expression was examined by Western blot (Figure 3A), and *frq* mRNA by qRTPCR (Figure 3B) in each SWI/SNF knockout at two times of day: circadian time (CT) 5 (DD16) when newly synthesized FRQ peaks and CT17 (DD28) when old FRQ is hyperphosphorylated and begins to be degraded [33]. In yeast, Snf5p, Swi1p, and Swi2p subunits are contacted by acidic activators such as Gcn4p and Hap4p [38]. Consistent with this, in the deletion strains examined, the FRQ and *frq* mRNA levels were low and circadian regulation was abolished in Δ*snf5*, Δ*swi1*, and decreased in Δ*swp59* (Figure 3). Consistent with WC-1 transcriptional autoregulation, the known role of FRQ in stabilizing WC-1 [4] and the low levels of FRQ in these strains, WC-1 levels were also correspondingly reduced, although it is also possible that SWI/SNF might directly influence WC-1 expression. Interestingly, although WC-1 levels in Δ*swi1* were slightly lower than in WT and higher than in Δ*swp82* (on the same blot), the Δ*swp82* strain had a normal FRQ level and circadian expression whereas Δ*swi1* did not (Figure 3A); this suggests that the very low WC-1 level in Δ*swi1* is sufficient to drive rhythmic *frq* expression and that the lack of a rhythm lies somewhere else. FRQ and WC-1 levels were similar to those of WT in other SWI/SNF knockouts examined (Figure S3A). Together, based on the low level of *frq* mRNA and FRQ in Δ*snf5*, Δ*swi1*, and Δ*swp59*, SWI/SNF complex participates in WCC-dependent *frq* expression and the SWI1 subunit is specifically involved in this event through its physical interaction with WC-1.

**Table 1:** *Neurospora* SWI/SNF subunits and knockouts.

Yeast subunits	Function	P value	*Neurospora* ortholog	Identity of gene deletion mutants in the whole genome knockout collection	Effect of gene deletion on FRQ expression
SWP59 (ARP9)	Actin related	1.6e^-16^	NCU08840	FGSC #18403	Reduced
				FGSC #18404	
SWP73 (SNF12)		5.7e^-14^	NCU03572	FGSC #18763	ND
SWI2 (SNF2)	Core subunit, ATPase activity	4.8e^-267^	NCU06488	FGSC #11467	ND
				heterokaryon	
SNF5	Core subunit	7.4e^-40^	NCU00421	FGSC #11785	Reduced
				FGSC #11786	
SWI1		4.9e^-21^	NCU05891	FGSC #11904	Reduced
				FGSC #11905	
SWI3	Core subunit	7.6e^-59^	NCU08003	FGSC #21999	ND
				heterokaryon	
SWP82		2.3e^-10^	NCU03064	FGSC #19756	ND
SWP29 (TAF14/TAF30)		2.3e^-46^	NCU00444	FGSC #18696	ND
				FGSC #18697	

*Neurospora* homologs were recovered based on similarity to Saccharomyces SWI/SNF subunits. All listed subunits are not essential in *Saccharomyces cerevisiae*. P value refers to the pBLAST score of the Neurospora ortholog when the yeast protein sequence was used to probe the Neurospora genome. In *Neurospora*, deletions of non essential genes are available as homokaryons whereas deletions of essential genes such as *swi2* and *swi3* are maintained as heterokaryons in which the deletion is mixed within syncytium with nuclei containing a WT copy of the gene.

### Rhythmic transcription of *frq* is abolished in Δ*swi1*

**Figure 4:**
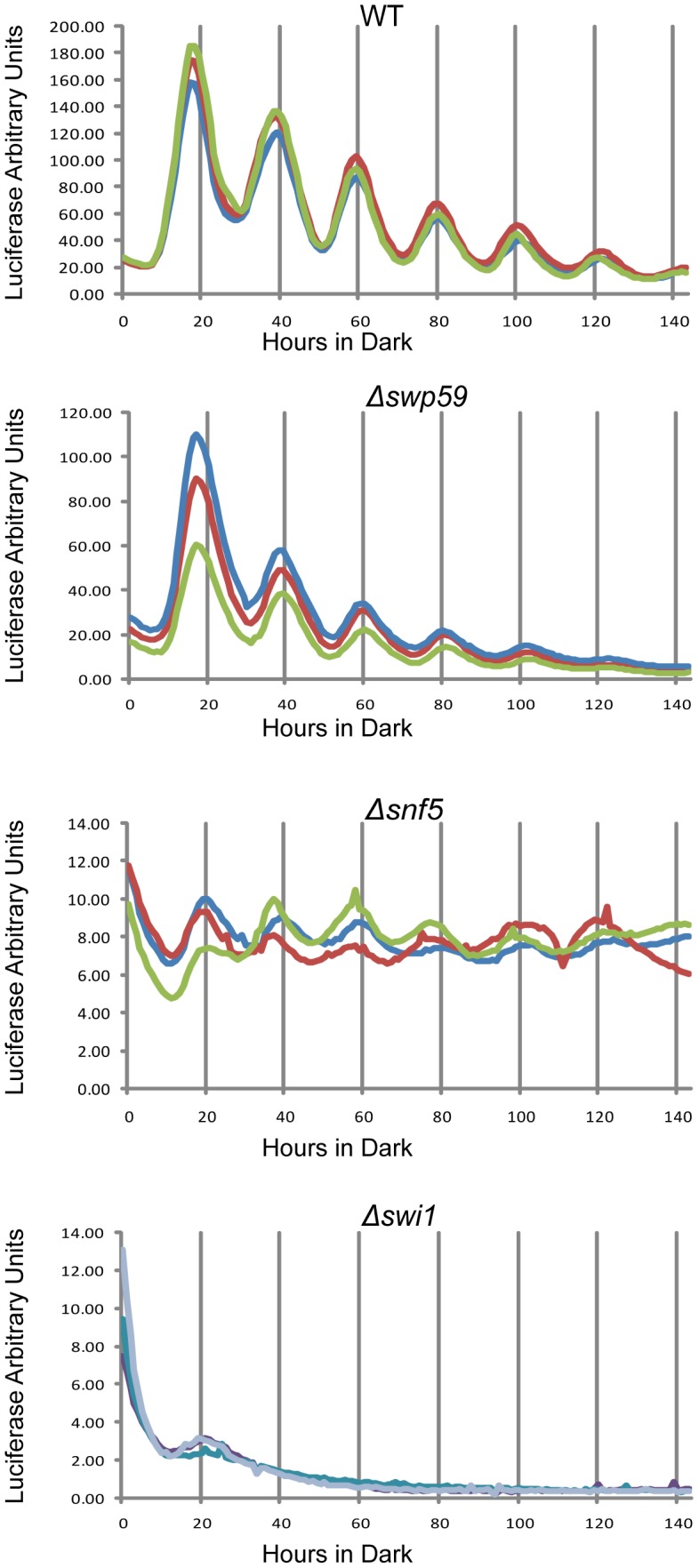
Reduced *frq* expression and loss of rhythmicity in SWI/SNF subunit knockouts as assayed by a luciferase reporter. *frq* transcription in WT, Δ*swi1*, Δ*snf5*, and Δ*swp59* was examined using the *frq C box* fused to codon-optimized firefly luciferase (transcriptional fusion). Strains were grown on 0.1% glucose racetube medium containing 5 mM luciferin in a 96 well plate and synchronized by growth in constant light for 48 hours followed by transfer to darkness. The luciferase signal was followed for longer than 6 days with sampling every 30 minutes. Each strain was repeated three times. Δ*swp59* showed a WT oscillation, Δ*snf5* also oscillated in a circadian manner despite an extremely low amplitube, while *frq* trancription was completely abolished in Δ*swi1*.

To check the circadian significance of *swi1*, *snf5* and *swi59*, the three deletion strains were backcrossed to the *ras-1^bd^* allele that has been widely used to visualize overt circadian rhythms in *Neurospora* [39]. In race tube assays Δ*swi59* showed a virtually WT period while, interestingly, both Δ*swi1* and Δ*snf5* were arrhythmic (Figure S3B), grew more slowly, and produced fewer conidia than WT, suggesting that SWI/SNF also plays a role in hyphal growth and asexual spore formation. The slowed growth and reduced conidiation phenotypes of Δ*swi1* and Δ*snf5* are consistent with those of yeast (see Introduction), but also raised the possibility that loss of rhythmicity was an artifact arising from interference of the mutations with the expression of rhythmicity rather than interference with the clock itself. To directly and continuously monitor changes in *frq* transcription at the level of the core clock, we created strains that bear an optimized *luciferase* reporter gene driven by the circadian promoter (*C box*) of *frq* [40] in Δ*swi1*, Δ*snf5*, and Δ*swi59* backgrounds respectively, such that the activity of the complex of WC-1 and WC-2 on the *frq* promoter could be analyzed *in vivo*. Consistent with Figure 3, the amplitude of *frq* promoter:luc transcription was dramatically impaired in Δ*swi1* while Δ*swi59* showed a WT phenotype (Figure 4). *frq* transcription was also impaired in Δ*snf5* but continued to oscillate weakly with a peak-to-trough amplitude only about 4% that of WT. The absence of the overt rhythm in this strain (Figure S3B) suggests that the severely attenuated rhythm in *frq* expression is not sufficient to drive the rhythms in *ccg* (*clock-controlled gene*) expression needed for the overt rhythm. Of particular note is that Δ*swi1* completely lost rhythmic transcription of *frq* and overall expression was about 1% of WT. Taken together, these data indicate that SNF5 contributes significantly to *frq* transcription whereas SWI1 is essential for the transcriptional oscillation of *frq* over a circadian cycle.

### The binding of SWI/SNF to the *C box* depends on aa 1-300 on WC-1

**Figure 5:**
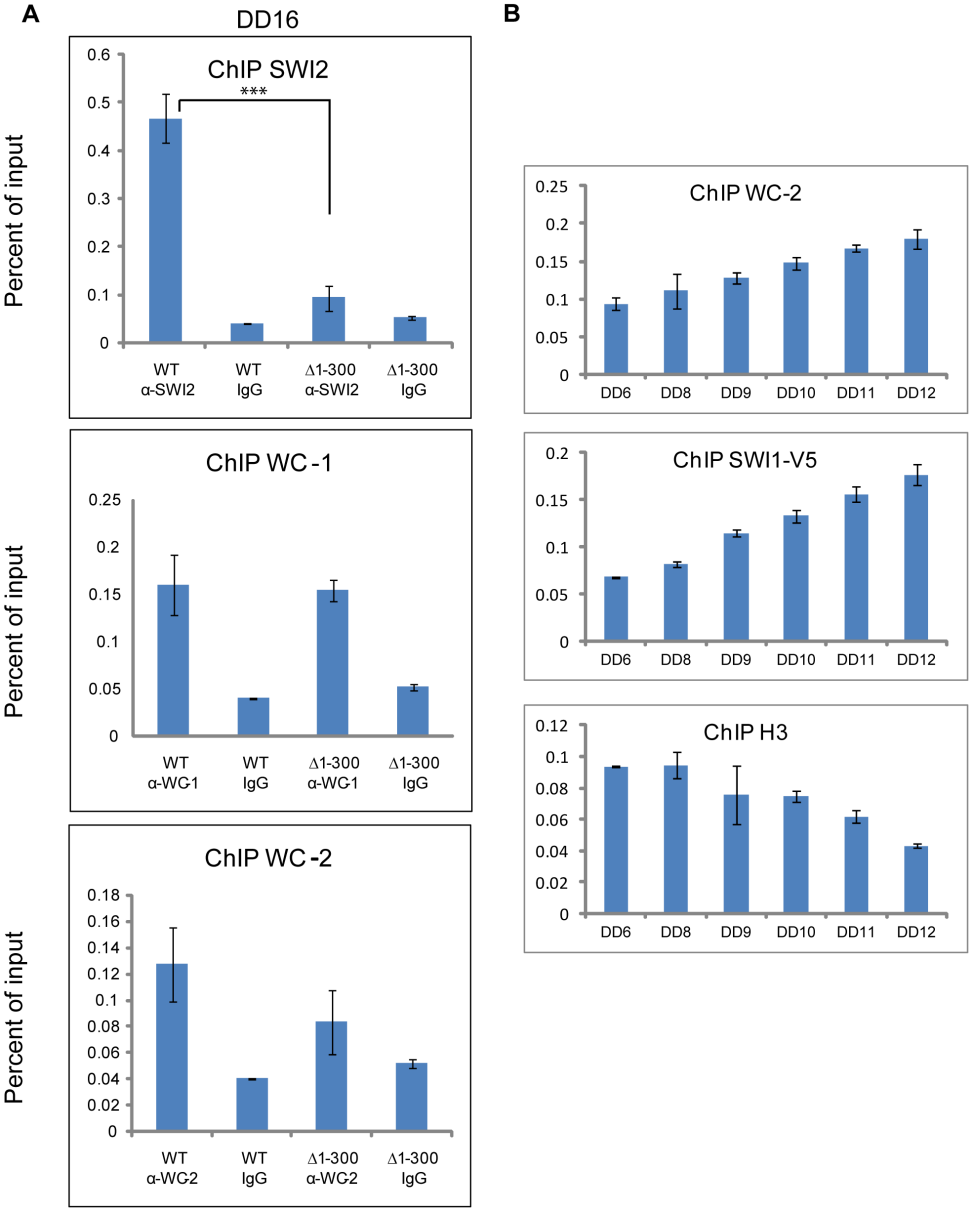
The binding of SWI/SNF to the *C box* relies on aa 1-300 of WC-1 and increases prior to the peak of *rq* expression. (**A**) ChIP experiment performed on chromatin isolated at DD16 when *frq* expression is maximal. WC-1 and WC-2 had a normal binding to the *C box* of *frq* in a strain bearing WC-1Δ1-300, while SWI2 binding was impaired in this strain. The annealing positions of the primer set used to detect the *C box* corresponds to the middle set shown in Figure 6A. Average values are plotted as a percent of total with error bars representing the standard error of the mean (SEM) (n = 3, ***p<0.0005). Samples were grown for 16 hours in the dark, formaldehyde-crosslinked, and harvested. (**B**) ChIP was used in a timecourse analysis of the association of WC-2, SWI-1 and histone H3 with the *C box*. Samples were gorwn for the indicated number of hours in darkness (DD) prior to harvesting and processing for ChIP as described in (A). Error bars represent the standard error of the mean ( = 3).

 Although the SWI/SNF complex remodels DNA and is required for the expression of some genes herein shown to include *frq*, it does not itself bind to DNA, but instead relies on dedicated transcription factors. Two reported models for SWI/SNF recruitment by transcription factors have been advanced: (1) Transcription factors that have a strong affinity for a promoter associate first with an Upstream Activation Sequence (UAS) and then recruit SWI/SNF [41], [42]; (2) those with weak binding to a UAS tend to recruit SWI/SNF off DNA first [41], [43], [44] and the bound SWI/SNF facilitates DNA association of these transcription factors (reviewed by 26). In *Neurospora*, WCC binds to the *PLRE* adjacent to the transcription start site (TSS) of the *frq* promoter under light conditions while it associates with the *C box* of the same promoter located ∼1.2 kilobases 5′ of the TSS (transcription start site) in the dark. To test whether SWI/SNF is recruited by WC-1 to the *C box*, in addition to determining which model fits best for WC-1, chromatin immunoprecipitation was performed at DD16, a time when *frq* expression should be near maximal, using the WT and WC-1 Δ1-300 strains (Figure 5A). SWI2, the core subunit of SWI/SNF, bound to the *C box* strongly in WT but did not bind to the *C box* in WC-1 Δ1-300; the binding of WC-1 and WC-2 to the same DNA sequence showed no significant difference in the two strains (Figure 5). The data suggest that WC-1 follows the first SWI/SNF model of binding in which the transcription factors bind strongly to the *C box*; recruitment of SWI/SNF is a subsequent event and not associated with binding of WC-2. A prediction of this model is that there should be a phased time-dependence to the association of these factors with the *C box* and their action on it. This was examined by ChIP in Figure 5B in a time series over the six hours leading up to maximal *frq* expression. Binding of the WCC (using WC-2 as a proxy) steadily increases over this period and this is accompanied by a slightly delayed and steeper increase in SWI/SNF association (using SWI1 as a proxy). Loss of nucleosome components from the region of the *C box* was tracked by loss of histone H3 which shows a lag of 3–4 hours followed by a rapid disappearance from the *C-box* region. A working model for initial events in activation of *frq* transcription consistent with these data posits WC-1 and WC-2 forming an active transcriptional complex on the *C box* DNA (see also [31]) followed by WCC recruitment of the SWI/SNF complex which initiates active remodeling of the chromatin in the region of the *C box*.

### Nucleosomal density at the *C box* of the *frq* promoter increases in Δ*swi1*

**Figure 6:**
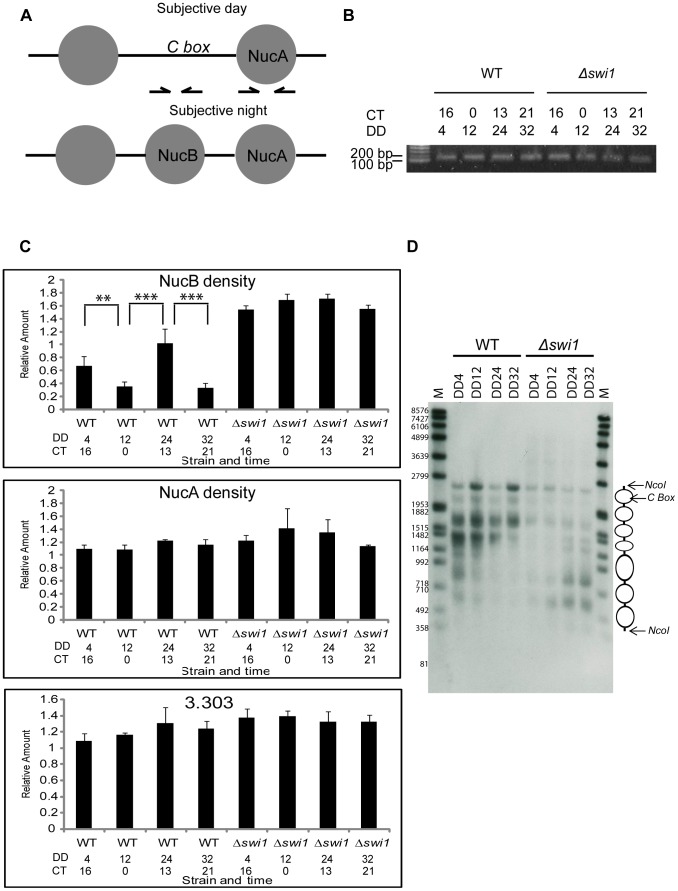
SWI1 is required for rhythmic opening of the *C box* through removal of NucB. (**A**) Relative positions of resident nucleosomes, NucB and NucA at the *C box* (see also reference 31). Arrows indicate primer sets used to detect these regions. (**B**) Nuclei were isolated by fractionation from samples harvested at indicated hours in the dark and digested with MNase. 20 nanograms of gel-purified mononucleosomal DNA were loaded in each lane. (**C**) The density of NucB was measured by real-time PCR using a primer set shown in (A, the left primer set) and gel purified mononucleosomal DNA as template (n = 3, error bars represent ± SEM, ***p<0.0005, **p<0.005). NucB occupancy of the *C box* oscillates between low (CT0 and 21) and high (CT 13-16) in WT as reported by Belden et al. (2007b), whereas NucB is always bound to the *C box* in Δ*swi*1. NucA and 3.303 (an unregulated control region of genomic DNA distinct from *frq*) served as controls for equal DNA inputs [31]. (**D**) Difference in *frq* promoter chromatin were probed with a limited nuclease digestion assay as described in Experimental Procedures. Cultures were harvested from WT or from Δ*swi1* cultures at the times in darkness indicated.

We have previously shown that a nucleosome (NucB) partially occludes the *C box* and blocks *frq* transcription in the subjective evening/night, and is removed from the *C box* in the late subjective night/early day when *frq* transcription is initiated [31] (Figure 6A); this model is consistent with the loss of histone H3 from the region as seen in Figure 5B. In the same study, an ATP-dependent chromatin remodeler, CLOCKSWITCH (CSW), was shown to be necessary for remodeling of the opened *C box* back to the closed repressive state. Our data suggested that SWI/SNF might be involved in antagonizing CSW in opening the *C box* for *frq* transcription. To compare NucB density between WT and Δ*swi1*, nuclei were isolated, micrococcal nuclease (MNase) digested, and mononucleosomal DNA was gel-purified and quantified for real-time PCR (Figure 6B). Four dark time points across two circadian cycles representing circadian oscillations of the NucB density were chosen for comparison: DD4 (CT 16, subjective night when the *C box* is closed and *frq* transcription repressed), DD12 (CT 0, subjective morning when the *C box* is open and *frq* is transcribed), DD24 (CT 13, subjective evening, second day), and DD32 (CT 21, late subjective night, second day); these times were chosen based on the peaks and troughs of nucleosome occupancy reported by Belden *et al*. [31]. NucB level was determined by real-time PCR using a specific primer set against DNA sequences near to the *C box* and the region of NucB on the mononucleosomal gel-purified DNA. As previously reported [31], NucB density peaked at DD4 and 24 and decreased at DD12 and 32 in WT (Figure 6C), but this oscillation was completely abolished in Δ*swi1* and NucB density was always higher than WT. To better gauge whether NucB was being moved aside or displaced to truly open up the chromatin at the *C box* we applied a nuclease sensitivity assay previously used to probe chromatin structure at this locus [31]. Chromatin was isolated from WT or Δ*swi1* strains at the same 4 subjective times (corresponding to two successive peaks and troughs in *frq* expression in WT and the corresponding times in Δ*swi* 1) and subjected to limited digestion with micrococcal nuclease (MNase), an enzyme that will cut open DNA but not DNA bound within nucleosomes (Figure 6D). The nucleosome located over the *C box* (NucB) appears to be rhythmically present in WT but continually present in the Δ*swi*1 background, consistent with the PCR analysis of Figure 6C. In all these data suggest that SWI/SNF is required for remodeling NucB to activate *frq* transcription in a circadian cycle and that SWI1 plays an essential role in this process. As controls for specificity, the nucleosome near NucB and bracketing the *C box* (called NucA, [31]) and an untranscribed region (3.303) of *Neurospora* DNA [31] had a comparable density between WT and Δ*swi1* across the four time points tested (Figure 6C).

## Discussion

In this study, we showed that WC-1 in the WCC recruits SWI/SNF to the *frq* promoter to aid in remodeling the nucleosome environment of the *C box* and thereby to initiate a circadian cycle of *frq* transcription. To identify potential transactivation domains on WC-1 required for *frq* transcription, a series of WC-1 deletions were generated and studied. Among these, all deletions covering amino acids 101-200 had normal WC-1 levels but displayed severely impaired *frq* mRNA and FRQ expression in the dark and arrhythmic circadian phenotypes. These data suggest aa 101-200 of WC-1 is a transactivation domain that is essential for circadian expression of *frq*. A search for coactivators recruited by WCC identified SWI/SNF, components of which interact with WC-1 *in vivo* and *in vitro*. In addition, the portion of WC-1 containing the transactivation domain is required for recruiting SWI/SNF to the *C box*.

Affinity purification of the *Neurospora* SWI/SNF complex identified the expected subunits based on yeast homology predictions (Table 1) as well as the protein encoded by NCU02006 (a highly conserved protein among fungi) and actin (that is also found in mammalian BAF or PBAF complex [45]–[49]). This complex is sturdy in yeast where deletion of the sites of activation domain contact in the N-terminal SNF5 and the second quarter of SWI1 left the SWI/SNF complex intact [50]. Assuming the Neurospora SWI/SNF complex is similarly robust the data suggest that the arrhythmic clock phenotype of Δ*swi1* and Δ*snf5* may be caused by the loss of transcription factor contact.

 A long standing question brought into focus by this study is how protein-DNA interactions at the *C box* bring about changes in *frq* transcription at the TSS, 1.2 kbp away. SWI/SNF, a complex with proven DNA looping capabilities [51], [52], provides a clear solution to this question through its ability to facilitate formation of DNA loops; these bring different genomic regions separated by kilobases into close proximity, resulting in sufficient concentrations of each transcription complex to drive transcription [53]. For example, Brg1 (SMCA4), a mouse SWI/SNF subunit, mediates compaction of chromatin into dense loops at the 200 kbp cytokine locus [54] and is also required for the formation of DNA loops across the 150 kbp CIITA locus during interferon-gamma (IFN-gamma)-mediated gene induction [55]. We anticipate that SWI/SNF recruited by the WCC remodels the *C box* region and brings about DNA looping in a similar manner to bring the *C box* into proximity with the TSS of *frq*.

 WCC is the primary blue light photoreceptor in the organism [1], [2], [9], acting at both the *C box* for circadian feedback and at the *PLRE* for acute light responses. Interestingly, while both SWI/SNF and aa 101-200 of WC-1 are indispensable for FRQ expression in the dark they have little influence on *PLRE*-mediated FRQ transcription in constant light. Light-induced FRQ was seen even in the WC-1 Δ1-300 strain; these data also explain the cryptic phenotype of the *rhy-2* strain arising from a WC-1 Δ1-264 [56]. WCC in the dark is mainly a heterodimer and when it senses light, it forms a multimer, the light active form, on the *PLRE* at the *frq* locus [2], [7], [57], [58]. Thus, it seems that WC-1 might recruit different coactivators in the light than in the dark to activate *frq* expression. One of the coactivators recruited by the light-activated WCC is NGF-1, a histone acetyltransferase, which plays a role in blue light signal transduction [59]. Also perhaps surprisingly the polyQ domains on the N- and C- termini of WC-1, long predicted to be transactivation domains, are needed for neither light nor dark activities of WC-1. This is consistent with previous work on N-polyQ polymorphisms in wild strains that showed only minor period differences [60], and with findings from other transcription factors such as yeast Gal4 that uses its acidic domain rather than glutamine-rich or proline-rich sequences to recruit SWI/SNF in transcription [27], [37], [38].

Through the lens provided by this study we can now begin to understand how multiple proteins and complexes with opposing functions coordinate their activities to open/activate and close/repress the *frq* locus at appropriate circadian phases. We previously reported Clock Switch (CSW) and CHD-1 as chromatin modifiers required for the rhythmic opening and closure of the *C box* in the *frq* promoter that leads to rhythmic expression of FRQ [31], [32]; CHD-1 also remodels the antisense *frq* (*qrf)* promoter and may play a more general role in maintaining chromatin structure at *frq* as without it expression levels never reach either peak or trough levels seen in WT [32]. WC-1, CSW, and CHD-1 are always present in the cell, and CSW appears to bind to the *C box* preferentially during the time when *frq* is becoming active [32]; without WC-1 *frq* is always inactive and without CSW or CHD-1 *frq* is always moderately active. In the core negative feedback loop, FRQ helps to inactivate WC-1 and prevents WCC from binding to DNA. However, the fact that *frq* remains moderately active without CSW or CHD-1 indicates that activation/repression of FRQ is more than only binding/inactivation of WCC but also requires the active participation of other factors, perhaps to eject the WCC/SWI/SNF complex from the *C box* and un-loop the DNA. Although both CSW and CHD-1 actively remodel DNA at *frq* their role in the clock cannot be succinctly stated as activating or repressing; the timing of CSW binding, for instance, coincides with *frq* activation yet *frq* is still expressed without it. When present and active, WCC might dominate the competition between activation and repression, recruiting SWI/SNF to activate *frq* despite the presence of other factors. Not explicitly accounted for yet in this model is the involvement of the *frq* antisense *qrf*, whose promoter is also remodeled by CHD-1. A recent publication revealed a novel factor, CATP (Clock ATPase), involved in remodeling chromatin at the *C box* [61]. The expression of *frq* in Δ*catp* strains appears to be about a log order greater than in strains lacking SWI/SNF, consistent with CATP acting as an accessory factor to help to open the *C box*.

**Figure 7:**
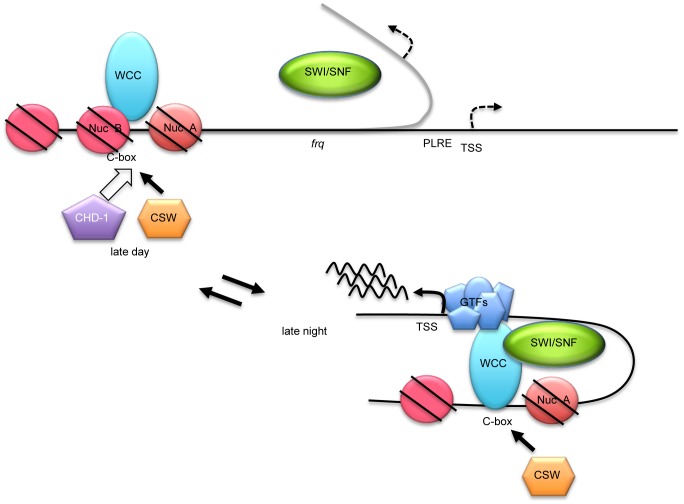
A working model for WC-1-dependent recruitment of SWI/SNF to initiate *frq* transcription. In the night before *frq* transcription starts, NucB is in a repressive state occluding the *C box* as fostered by chromatin remodelers that may include CHD-1. When active WC-1/WC-2 begin to bind at the *C box*, an event roughly coincident with CSW binding, SWI/SNF is recruited and it helps to fully remodel the *C box* to an active state, removing NucB, and also beginning to bring about the DNA looping (curved gray line) that is essential to bring the WCC transcriptional activation domains to the transcription start site (TSS) for stable recruitment of general transcription factors (GTFs). For acute light-induction of *frq*, an action not requiring SWI/SNF, WCC binds directly to the *PLRE* rather than the *C box* and recruits unknown remodelers to drive *frq* transcription at levels much higher than in the dark.

A working model based on these data is summarized in Figure 7. In the dark, before *frq* transcription starts, NucB mostly blocks the *C box* due to the action of chromatin remodelers that may include CHD-1; when with WC-2, WC-1 binds to the *C box* in its active form, it recruits SWI/SNF which aids in removing NucB from the *C box*, stabilizing the active state essential for *frq* transcription; CSW binds most strongly at this time. Based on the action of SWI/SNF in other systems we anticipate that its action involves DNA looping to bring the TSS into proximity with the *C box* and WCC. This configuration remains active, with active WC-1 being rapidly turned over, until FRQ depresses activity of WC-1 and reduces its affinity for DNA thereby also stabilizing it. Without WCC bound, the SWI/SNF-mediated looping is reversed by the action of other chromatin remodelers, and NucB returns to cover the *C box*.

 A variety of data suggest that light activation of *frq* may represent a distinct function. In the light *frq* mRNA levels are much higher than that in the dark, FRQ does not inhibit this expression, [33], [62] and in the light, the FRQ levels are nearly normal in the SWI/SNF deletion strains tested including Δ*swi1*, Δ*snf5* and Δ*swp59* that severely reduce circadian *frq* expression. This suggests that the WCC recruited to the *PLRE* in the light recruits functional coactivators other than SWI/SNF to modify the *PLRE* in *frq* transcription. Additionally and consistent with this, the *PLRE* is located adjacent to the TSS site and WCC acts on these regions neither by looping of the DNA to bring activators to the TSS, nor by wholesale remodeling of chromatin, although epigenetic modifications have been noted [32], [39], [59]. This suggests that major remodeling and looping induced by SWI/SNF are principal factors distinguishing circadian activators at the *C box* versus light activators at the *PLRE*.

## Materials and Methods

### Strains and growth conditions

328-4 (*ras-1^bd^ A*) and 74A (*ras-1^WT^ A*) were each used as a clock-WT strain in this study. Race tube analyses were carried out as previously described [39]. Race tube medium contains 1×Vogel's salts, 0.1% glucose, 0.17% arginine, 50 ng/mL biotin, and 1.5% bacto-agar, and liquid culture medium (LCM) is 1×Vogel's, 0.5% arginine, and 50 ng/mL biotin with glucose at 2%. Race tubes were inoculated and incubated in constant light for 16–24 h at 25°C and then transferred to constant darkness at 25°C. A recipient strain for generating WC-1 deletion series is 21-9 (*ras-1^bd^; Δfrq::hph^+^; Δmus-52::hph^+^ a*). *Neurospora* transformation was done as previously described [63]. The *wc-1* {knock-in} (*wc-1^KI^*) targeting cassette pWB-1-6 was introduced into 21-9 and replacement mutants were backcrossed to 328-4 to obtain homokaryotic strains for race tube analyses. WC-1 and SWI/SNF deletion strains generated by the *Neurospora* genome project were obtained from Fungal Genetics Stock Center (FGSC) [63].

### Protein isolation and detection

Procedures for preparation of protein lysates and Western blots were followed as described [33], [64]. For Western blot, 15 milligrams of whole-cell protein lysate was loaded per lane. Anti-V5 antibody (Pierce) was diluted 1∶5000 for use as the primary antibody. SWI2 antibody was obtained from Abcam (Ab3749). Protein purification prior to MS/MS analysis was performed using a slightly modified procedure [64].

#### Immunoprecipitation (IP)

IP was done as previously described [64]. In brief, 2 milligrams of total protein were incubated with 50 µL of V5 beads rotating for 2 hours to overnight. The agarose beads were washed with the protein extraction buffer 4 times and eluted with 50 uL of 5× SDS sample buffer at 99°C for 5 min.

### GST pull-down assay

WC-1 aa1-300 and 100-300 were each cloned into pGEX4T1 in-frame fused with an N-terminal GST tag, and a hexahistidine tag was added by PCR to the C-termini. The plasmids were expressed in bacteria grown in LB medium with Ampicillin at a concentration of 10 ug/mL for 3 hours and induced with 1 mM IPTG for 1 hour. For WC-1 aa1-300 and 100-300, a cobalt purification step (Pierce) followed by a subsequent glutathione (Thermo Pierce) step was carried out to obtain full length polypeptides. GST was purified with glutathione resin (Thermo Pierce) alone. *Neurospora* lysates were cleared by centrifugation at 9,000 g for 10 minutes at 4°C. Purified GST, GST-aa1-300, and aa100-300 on glutathione beads were each incubated with 2 milligrams of cleared *Neurospora* lysate (SWI1 C-tagged with V5) and rotated for at least 2 hours. The supernatant was removed and the beads were washed three times with the pull-down buffer.

### Micrococcal nuclease assays

The micrococcal nuclease assay was performed as described in [31] with modifications. In brief, *Neurospora* nuclei were isolated from tissues cultured for indicated dark time. For each sample, 80 µgs of nuclei were digested with micrococcal nuclease (Takara) at the final concentration of 0.1 unit/ml for 1.5 min at 37 degrees. The digestion reaction was stopped by adding a buffer containing 0.2 mg/ml protease K and incubated at 37 degrees overnight. Chromatin DNA was extracted using Gentra Puregene Cell Kit (Qiagen) and cut with *Nco*I. Southern blot was carried out with a digoxigenin-labeled probe as described in [31].

### Other techniques

Chromatin immunoprecipitation experiments were done as previously described [31]. Mass Spectrometry was performed as previously described [64]. Luciferase assays were performed as previously described [40]. Nuclear preparations and MNase digestions were performed as reported [31], [65].

## Supporting Information

Figure S1 Race tube phenotypes of *wc-1* mutants between aa 100-200 and FRQ protein levels in Δ163-200 (**A**) Amino acid sequence alignment of four fungal WC-1s. When WC-1 sequences from four closely related fungi (*Neurospora crassa*, *Magnaporthe grisea, Podospora anserina,* and *Chaetomium globosum*) were aligned, it is clear that the polyQ domains are not conserved. Amino acid sequences were downloaded from the NCBI website and the alignments were performed using the EMBI-EBI on-line tool ClustalW2 (http://www.ebi.ac.uk/Tools/msa/clustalw2/) (**B**) Race tube analyses of *wc-1* mutants in the region of aa 100-200. 328-4 (*ras-1^bd^*) was WT for this assay. Period lengths were as shown +/- SEM. (**C**) Western blot analysis of FRQ and WC-1 in WT and WC-1 Δ163-200 in constant light, DD16, and DD28. (**D**) FRQ is normally induced in response to light in Δ163-200. After a light exposure of 15 minutes, WC-1 underwent hyperphosphorylation and light-induced FRQ was seen after 1 hour light pulse in WT and Δ163-200.
(TIF)

Figure S2 Protein levels of WC-1, WC-2, SWI1, and SWI2 in constant dark over one circadian cycle. WC-1, WC-2, SWI1, and SWI2 protein levels were examined by Western blot across 28 hours in the dark. WC-1, WC-2, SWI1, and SWI2 showed relatively even protein levels. 15 µgs of total protein lysate were loaded into each lane.
(TIF)

Figure S3 FRQ expression in SWI/SNF single subunit knockouts and race tube phenotypes of Δ*swi1*, Δ*snf5*, and Δ*swp59* (**A**) FRQ and WC-1 levels in SWI/SNF deletion strains analyzed by Western blot. Samples were grown in 2% LCM medium, synchronized in the light, transferred to the dark, and harvested at indicated time points. Non-specific bands were shown to demonstrate equal loading. (**B**) Δ*swi1*, Δ*snf5*, and Δ*swp59* strains with and without the *ras-1^bd^* mutation grown on standard race tube medium. Black lines marked daily growth fronts of the strains in race tubes. Δ*swi1* and Δ*snf5* in the *ras-1^bd^* background displayed conidiation and growth defects.
(TIF)
