# Supplementary figures and images for: Neurospora WC-1 Recruits SWI/SNF to Remodel frequency and Initiate a Circadian Cycle
Source: PLoS Genet. 2014 Sep 25;10(9):e1004599. doi: 10.1371/journal.pgen.1004599 (PMC4177678; doi:10.1371/journal.pgen.1004599)

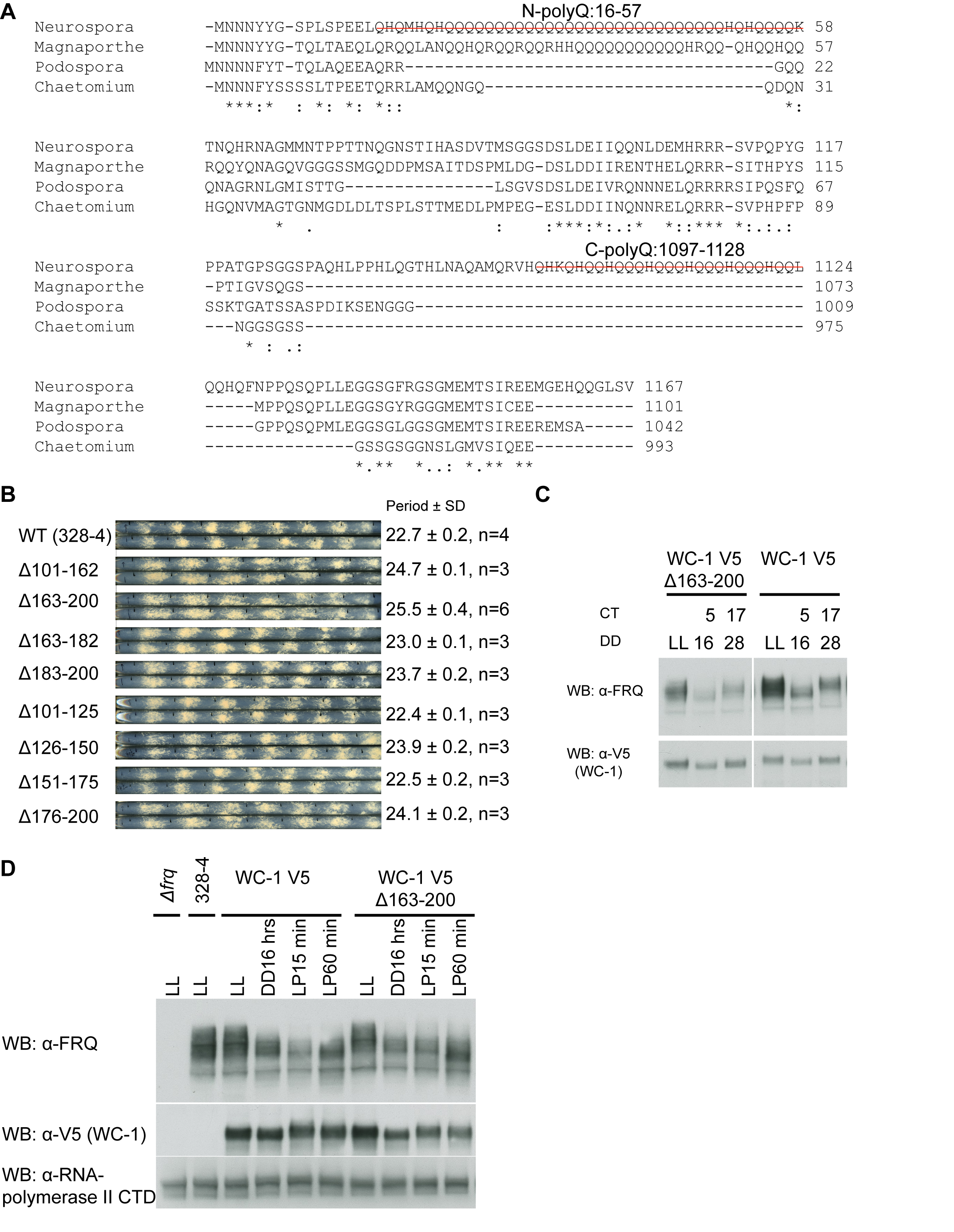

Supplement: Figure S1 — Race tube phenotypes of wc-1 mutants between aa 100-200 and FRQ protein levels in Δ163-200 (A) Amino acid sequence alignment of four fungal WC-1s. When WC-1 sequences from four closely related fungi (Neurospora crassa, Magnaporthe grisea, Podospora anserina, and Chaetomium globosum) were aligned, it is clear that the polyQ domains are not conserved. Amino acid sequences were downloaded from the NCBI website and the alignments were performed using the EMBI-EBI on-line tool ClustalW2 (http://www.ebi.ac.uk/Tools/msa/clustalw2/) (B) Race tube analyses of wc-1 mutants in the region of aa 100-200. 328-4 (ras-1bd) was WT for this assay. Period lengths were as shown +/- SEM. (C) Western blot analysis of FRQ and WC-1 in WT and WC-1 Δ163-200 in constant light, DD16, and DD28. (D) FRQ is normally induced in response to light in Δ163-200. After a light exposure of 15 minutes, WC-1 underwent hyperphosphorylation and light-induced FRQ was seen after 1 hour light pulse in WT and Δ163-200. (TIF) [file pgen.1004599.s001.tif]

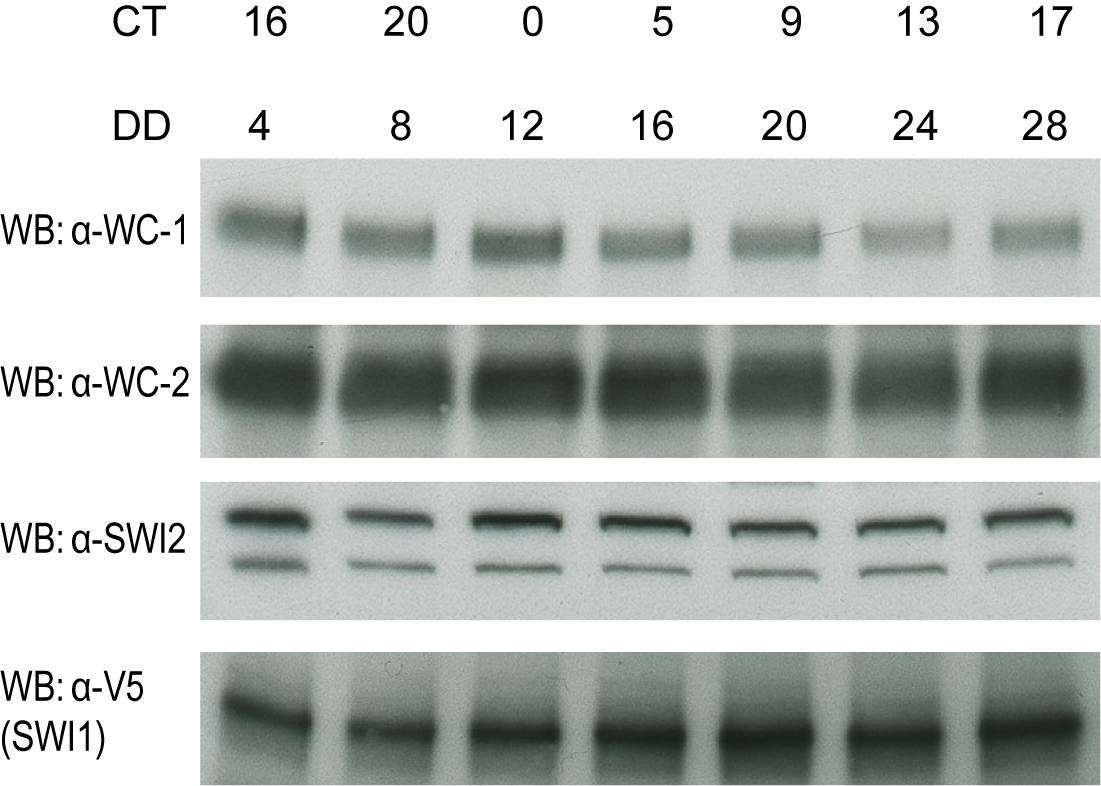

Supplement: Figure S2 — Protein levels of WC-1, WC-2, SWI1, and SWI2 in constant dark over one circadian cycle. WC-1, WC-2, SWI1, and SWI2 protein levels were examined by Western blot across 28 hours in the dark. WC-1, WC-2, SWI1, and SWI2 showed relatively even protein levels. 15 µgs of total protein lysate were loaded into each lane. (TIF) [file pgen.1004599.s002.tif]

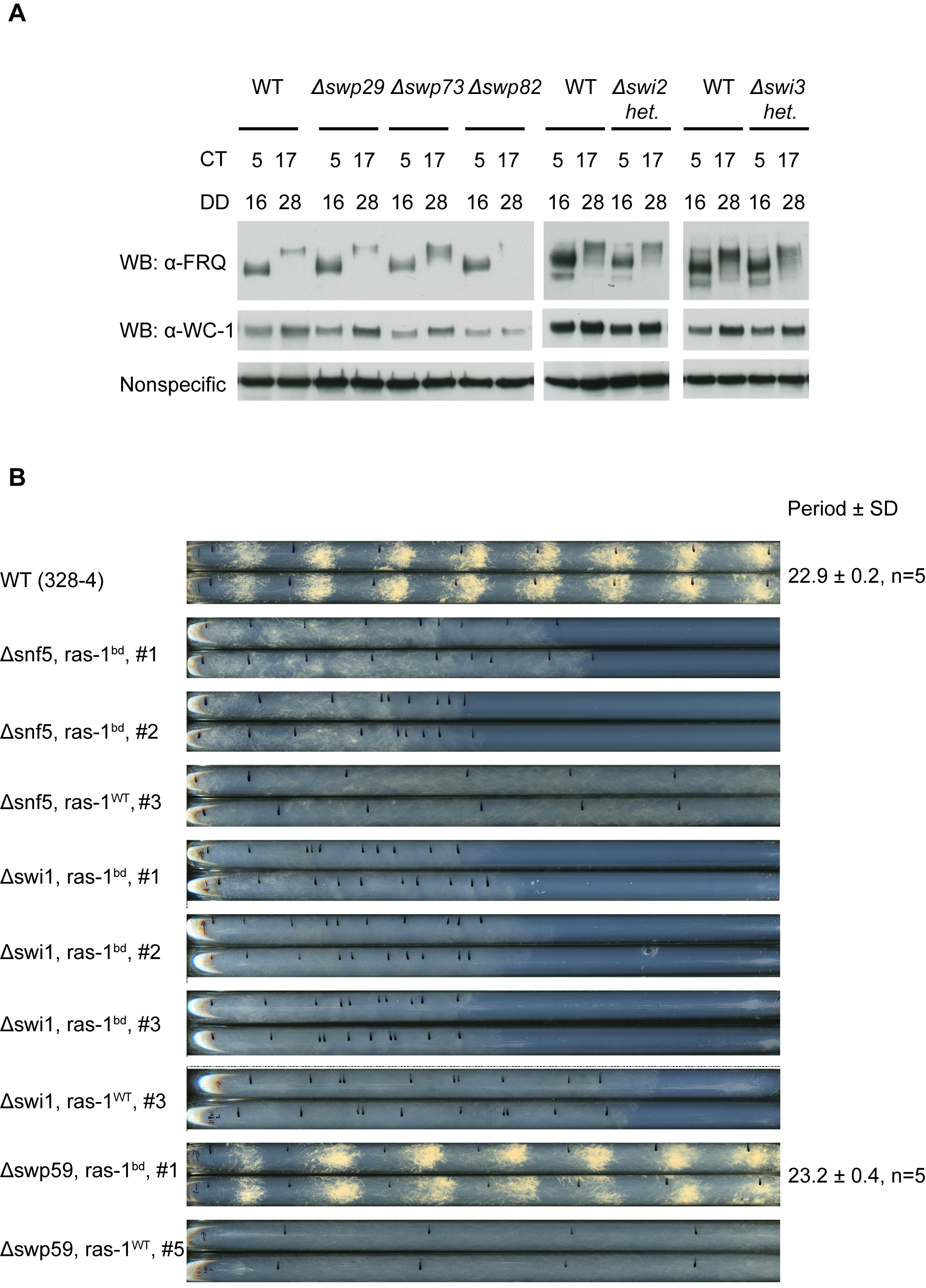

Supplement: Figure S3 — FRQ expression in SWI/SNF single subunit knockouts and race tube phenotypes of Δswi1, Δsnf5, and Δswp59 (A) FRQ and WC-1 levels in SWI/SNF deletion strains analyzed by Western blot. Samples were grown in 2% LCM medium, synchronized in the light, transferred to the dark, and harvested at indicated time points. Non-specific bands were shown to demonstrate equal loading. (B) Δswi1, Δsnf5, and Δswp59 strains with and without the ras-1bd mutation grown on standard race tube medium. Black lines marked daily growth fronts of the strains in race tubes. Δswi1 and Δsnf5 in the ras-1bd background displayed conidiation and growth defects. (TIF) [file pgen.1004599.s003.tif]
